# An improved understanding of pediatric chronic nonbacterial osteomyelitis pathophysiology informs current and future treatment

**DOI:** 10.1093/jbmr/zjae141

**Published:** 2024-08-29

**Authors:** Eve Roberts, Amandine Charras, Gabriele Hahn, Christian M Hedrich

**Affiliations:** Department of Women's & Children's Health, Institute of Life Course and Medical Sciences, University of Liverpool, Liverpool, United Kingdom; Department of Women's & Children's Health, Institute of Life Course and Medical Sciences, University of Liverpool, Liverpool, United Kingdom; Department of Pediatric Radiology, University Children’s Hospital Basel UKBB, Basel, Switzerland; Department of Women's & Children's Health, Institute of Life Course and Medical Sciences, University of Liverpool, Liverpool, United Kingdom; Department of Paediatric Rheumatology, Alder Hey Children's NHS Foundation Trust Hospital, Liverpool, United Kingdom

**Keywords:** CNO, CRMO, osteomyelitis, treatment, inflammation, bone

## Abstract

Chronic nonbacterial osteomyelitis (CNO) is an autoinflammatory bone disease that primarily affects children and young people. It can cause significant pain, reduced function, bone swelling, and even (vertebral body) fractures. Because of a limited understanding of its pathophysiology, the treatment of CNO remains empiric and is based on relatively small case series, expert opinion, and personal experience. Several studies have linked pathological NOD-kike receptor (NLR) family pyrin domain containing 3 (NLRP3) inflammasome activation and the resulting imbalance between pro- and anti-inflammatory cytokine expression with CNO. This agrees with elevated pro-inflammatory (mostly) monocyte-derived protein signatures in the blood of CNO patients that may be used as future diagnostic and/or prognostic biomarkers. Recently, rare variants in the *P2RX7* gene, encoding for an ATP-dependent transmembrane channel, were linked with increased NLRP3 inflammasome assembly and prolonged monocyte/macrophage survival in CNO. Although the exact molecular mechanisms remain unclear, this will inform future target-directed and individualized treatment. This manuscript reviews most recent developments and their impact on diagnostic and therapeutic strategies in CNO.

## Introduction 

**Figure 1:**
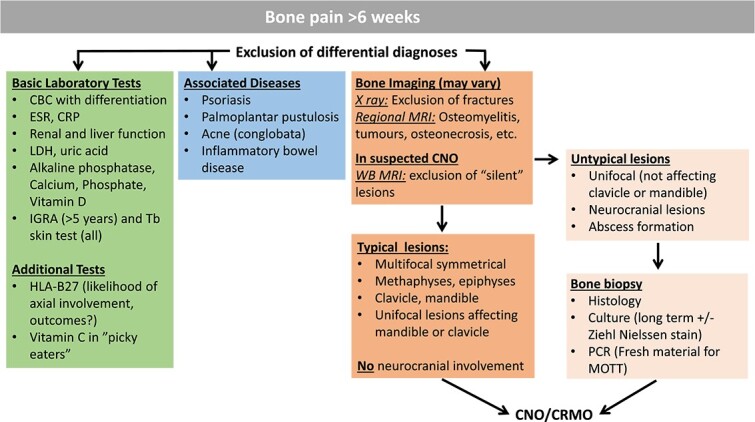
Diagnostic approach in CNO. Abbreviations: CRP, C-reactive protein; ESR, erythrocyte sedimentation rate; LDH, lactate dehydrogenase; IGRA, interferon gamma release assay; Tb, tuberculosis; HLA, human leucocyte antigen.

Chronic nonbacterial osteomyelitis (CNO) is an auto-inflammatory bone disease characterized by sterile bone inflammation, resulting in osteolytic and/or osteosclerotic bone damage.^1^^,^^2^ Although the majority of publications focus on children and young adults,^2^ CNO also affects adults and may be underreported in this age group.^3^ Inflammatory bone lesions result in pain, swelling, and loss of function, with some patients experiencing long-term complications such as vertebral compression fractures, bone overgrowth (most commonly affecting the clavicle or mandible), and/or arthritis (Figure 1).^4^^,^^5^ Thus, CNO significantly impacts the quality of life and wellbeing of affected patients, impacting school attendance and educational choices, relationships, and finances.^6^

The exact incidence of CNO (across age groups) remains unclear. Recent results from a British Pediatric Surveillance Unit study estimated 0.605 pediatric CNO cases per 100 000 person years.^7^ However, in part due to limited awareness and its variable and nonspecific clinical picture, CNO is likely underdiagnosed across age groups.^3^^,^^5^^,^^8^^,^^9^ Indeed, a single-center study from Germany suggested significantly higher regional prevalence of CNO that was similar to infectious osteomyelitis in children (estimated 80/100 000).^5^

This manuscript summarizes key clinical presentations and complications, and reviews recent developments in understanding disease pathomechanisms that inform current and future treatment with a focus on pediatric CNO.

### Clinical picture

CNO can present as a spectrum of clinical features ranging from single unifocal lesions in some to multifocal chronically active or recurrent bone inflammation in the majority of patients, also referred to as chronic multifocal recurrent osteomyelitis (CRMO).^8^^,^^10^ Although the majority of published reports focus on children and young people with CNO (average age at onset in pediatric CNO: 7–12 yr),^2^^,^^5^^,^^11^ adults can also be affected and are likely underreported.^3^^,^^12^ The clinical picture of CNO in adults may differ from children with fever involvement of long bones of extremities, but more axial skeletal inflammation.^3^^,^^12^ Furthermore, although relatively rare in children, a significant proportion of adults with CNO exhibit additional features, including synovitis, acne, (palmoplantar) pustulosis, hyperostosis, and osteitis, which is also referred to as SAPHO.^3^^,^^12–17^ Notably, SAPHO is thought to be closely related to CNO (indeed, recognized as part of the clinical spectrum of CNO in adults^3^^,^^12^) and may result from prolonged untreated immune activation in CNO.^17^^,^^18^

Several inflammatory diseases are associated with CNO, including inflammatory bowel disease (IBD), in approximately 10% of CNO patients,^5^^,^^19^^,^^20^ cutaneous manifestations (18%–21%,^5^^,^^21^^,^^22^), including psoriasis which may be more prevalent in patients treated with TNF inhibitors (TNFi),^5^^,^^23^ severe acne, Sweet syndrome and pyoderma gangrenosum, and Takayasu arteritis.^24–27^ Recently, pulmonary involvement, affecting between 3% and 8% of patients, has been reported in a national cohort from Germany.^27^

### Diagnostic approach and differential diagnoses

Currently, CNO remains a diagnosis of exclusion that sometimes requires bone biopsies to rule out differential diagnoses, such as infections (bacterial or fungal osteomyelitis, mycobacterial infections, actinomycosis), metabolic bone disease (hypophosphatasia),^28^ benign bone tumors (osteoid osteoma, osteoblastoma, fibrous dysplasia, enchondromatosis, hemangiomatosis, etc.), hematological (leukemia, lymphoma) or solid (Ewing sarcoma, osteosarcoma, secondary bone metastases, primary intraosseous lymphoma) malignancy, other autoinflammatory diseases with bone involvement (Pyogenic Arthritis, Pyoderma gangrenosum, and Acne, deficiency of the IL–1 receptor antagonist (DIRA), Majeed syndrome),^1^^,^^10^^,^^11^^,^^29^^,^^30^ and others (Osteonecrosis, Langerhans cell histiocytosis, immunodeficiencies, neuroblastoma, hypertrophic osteoarthropathy, scurvy, bone bruise/trauma, transient osteoporosis, (stress) fractures, etc.) (Figure 1). Two sets of classification criteria have been suggested (Table 1).^9^^,^^31^ Both criteria were generated from a retrospective review of CNO/CRMO cases presenting with a range of clinical features, covering a spectrum from single inflammatory lesions to multifocal recurrent disease.^9^^,^^31^ However, case numbers were relatively small, and neither group tested the criteria against differential diagnoses (other than bacterial osteomyelitis in^31^), while prospective validation in independent cohorts is lacking. As a result, internationally agreed and independently validated diagnostic or classification criteria for CNO are currently lacking. A large cohort of 450 cases from 7 different countries, including CNO/CRMO and “mimicker conditions,” have recently been reviewed by members of the American College of Rheumatology (ACR) and the European Alliance of Associations for Rheumatology (EULAR).^32^ Based on results from this exercise and following a stringent expert consensus exercise, the collaborative group developed candidate classification criteria that have been recently shared at the ACR 2022 Annual Meeting, with final dissemination awaited.

**Table 1:** Comparison of suggested classification criteria for CNO/CRMO.^9^^,^^31^

	**Roderick et al.** ^9^	**Jansson et al.** ^31^
**Case numbers**	41 patients	89 patients
**Time period studied**	2005-2012	1997-2005
**Diagnostic criteria**	Presence of typical clinical findings AND Presence of typical radiological findings AND EITHER Criterion 1: more than one bone (or clavicle alone) without elevated CRP (<30 g/L) OR Criterion 2: whether unifocal disease (other than clavicle), or CRP >30 g/L, with bone biopsy showing inflammatory changes with no bacterial growth while not on antibiotic therapy	Presence of 2 major criteria or 1 major and 3 minor criteria: Major criteria: Radiologically proven osteolytic/-sclerotic bone lesion Multifocal bone lesions PPP or psoriasis Sterile bone biopsy with signs of inflammation and/or fibrosis, sclerosis Minor criteria: Normal blood count and good general state of health CRP and ESR mildly to moderately elevated Observation time longer than 6 mo Hyperostosis Associated with other autoimmune diseases apart from PPP or psoriasis Grade I or II relatives with autoimmune or autoinflammatory disease, or with NBO
**Aim**	To explore the criteria used for diagnosis with and without biopsy	To distinguish CNO from other disease entities with similar clinical presentations To diagnose patients with osteitis alone
**Sensitivity**	34/41 (82.9%) patients diagnosed by criterion 1 alone 14.6% of children would require bone biopsy 1 child did not meet criteria for diagnosis	All children^33^ met criteria for diagnosis. The more criteria that were met, the more likely a CRMO diagnosis was made

Abbreviations: ESR, erythrocyte sedimentation rate; CNO, chronic nonbacterial osteomyelitis; CRMO, chronic multifocal recurrent osteomyelitis; CRP, C-reactive protein; PPP, Palmoplantar pustulosis

**Figure 2:**
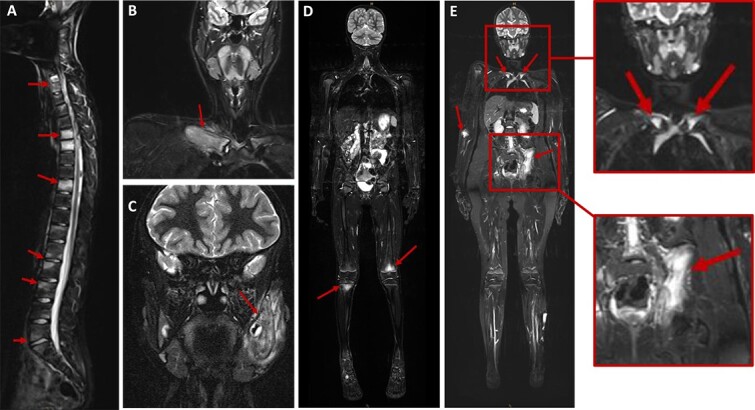
MRI findings in patients with CNO. Whole body (WB) MRI can detect inflammation before structural changes are present. (A) Sagittal TIRM sequences showing total and focal hyperintensities in multiple vertebral bodies. (B) Coronal TIRM sequences with hyperintense thickening of the left mandible and surrounding inflammatory soft tissue. (C) Coronal TIRM sequences revealing hyperintense thickening of the right clavicle and surrounding inflammatory soft tissue. (D) TIRM-sequences showing typical hyperintense lesions in the distal femoral metaphysis (right) and proximal tibial metaphysis (left). (E) TIRM-sequences revealing CNO-typical hyperintense lesions in the distal humeral metaphysis (right), in the medial claviculae and os ilium (left). Boxes on the right show enlarged sections displaying inflammatory activity affecting with claviculae (top) and os ilium (bottom).

Imaging techniques are central diagnostic tools in diagnosing and monitoring treatment response in CNO/CRMO. Plain X-rays are often used early in the diagnostic process and may show osteosclerosis, osteolysis, cortical thickening, and/or fractures.^34^^,^^35^ However, isolated bone edema in the absence of structural damage will be missed on plain films. Whole body MRI has become the “gold standard” imaging technique to diagnose and monitor CNO/CRMO (Figure 2). It can detect inflammation before structural changes are present through signal alterations of bone marrow or surrounding soft tissues on heavily T2-weighted fat-saturated sequences (STIR or TIRM) (Figure 2).^36^^,^^37^ Furthermore, whole body (WB) MRI allows for the detection of clinically quiescent lesions, which may inform treatment.^38^^,^^39^ In cases where the use of WB-MRI is contraindicated or not accessible, serial MRIs covering multiple body parts or (in exceptions) bone scintigraphy may be used. However, scintigraphy comes with the disadvantage of significant exposure to radiation while being less sensitive when compared to MRI, and enhancement of growth plates may mask adjacent inflammatory lesions.^40^ Notably, MRI uses no ionizing radiation, making it a preferable imaging modality in pediatrics. The use of conventional CT or ultrasound for diagnosing CNO is diminishing with improved knowledge of the condition; however, these methods may be useful in the initial stages of diagnosis to rule out other causes, such as malignancy, and to look for soft-tissue pathology (ultrasound).^35^

Although not playing a role in pediatric CNO (mainly because of exposure to radiation), PET/CT can be helpful to diagnose and monitor patients with adult SAPHO/CNO, where chest wall involvement may not be sufficiently represented on MRI (because of motion artefacts).^41^^,^^42^ Notably, scintigraphy of the sternoclavicular region may show a “bull’s head” pattern of radionuclide uptake that is deemed pathognomonic for SAPHO in the adult age group.^42^

### Pathophysiology

Until recently, the molecular pathophysiology of CNO/CRMO remained unclear. Recent laboratory studies have provided valuable insights, explaining the efficacy of currently used treatments while suggesting future targeted interventions. Based on their historic definition as systemic inflammatory diseases in the absence of an external trigger, no high-titer autoantibodies, or auto-reactive lymphocyte involvement, CNO has been classified as an autoinflammatory disease.^43–46^ Indeed, innate immune cells from CNO patients, namely monocytes, are characterized by an inflammatory molecular phenotype with reduced expression of immune regulatory signals and increased expression and release of pro-inflammatory cytokines.^46–50^

### Reduced expression of immune regulatory cytokines

**Figure 3:**
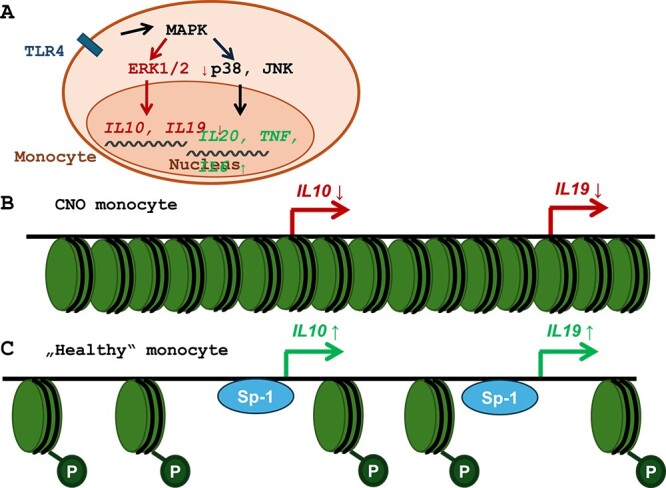
Molecular mechanisms of imbalanced cytokine expression in CNO. (A) Altered phosphorylation (activation) of ) in response to TLR 4 stimulation (with LPS) is a hallmark of CNO monocytes. Although «other» MAPKs (including p38, JNK) are activated normally, MAPKs ERK1/2 fail to be phosphorylated, which contributes to reduced expression of immune-regulatory IL–10 and IL–19. Reduced ERK1/2 activity results in impaired phosphorylation and nuclear shuttling of the transcription factor Sp-1 and altered phosphorylation of histone H3 at position serine 19 (H3S19p), an «opening» epigenetic mark. This, in summation, results in *IL10* and *IL19* «compaction» and reduced gene expression in CNO monocytes (B) when compared to healthy controls (C).

Interleukins (IL)-10 and IL–19 have immune regulatory functions, including the inhibition of pro-inflammatory responses of both the innate and the adaptive immune cells, the suppression of pro-inflammatory cytokine expression, Toll-like receptor (TLR) signaling, and inflammasome assembly.^2^^,^^46^^,^^51–53^ Impaired expression of IL–10 and IL–19 has been observed in monocytes from patients with CNO/CRMO^46^^,^^48^^,^^52^ (Figure 3A). This is the result of altered activation of MAPKs ERK 1 and 2 in response to TLR 4 activation with LPS,^48^^,^^49^ which results in reduced phosphorylation of the transcription factor signaling protein (SP-)1, a key activator of IL–10 and IL–19 expression^49^ (Figure 3). Furthermore, reduced ERK1/2 activation contributes to reduced phosphorylation of histone H3 at serine 10 (H3S10p) affecting the *IL10* and *IL19* promoters on chromosome 1.^48^ Histone H3S10p is an “opening” epigenetic mark that allows transcription factors and RNA polymerases to access regulatory regions. Thus, reduced nuclear SP-1 and limited promoter accessibility result in altered gene expression.^49^ Notably, the pro-inflammatory IL–10 family cytokine IL–20 is not affected and normally expressed (alongside IL–6 and TNF) (Figure 3A).^49^

### Increased inflammasome expression and assembly, and pro-inflammatory cytokine release

**Figure 4:**
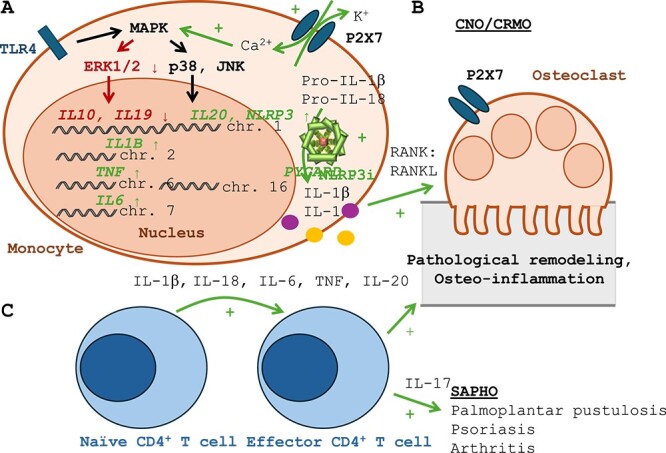
CNO-associated immune dysregulation results in pathological bone remodeling and inflammatory bone loss. (A) Altered MAPK activation in CNO monocytes contributes to reduced expression of immune regulatory IL–10 and IL–19, while pro-inflammatory cytokine and inflammasome component expression remains normal (or are increased). CNO-associated variants in P2X7 affect NLRP3 inflammasome assembly through increased potassium (K^+^) efflux. Subsequent caspase-1 activation results in increased IL–1β and IL–18 release. (B) The resulting imbalance between pro- and anti-inflammatory signals contributes to increased interactions between RANK and its ligand (RANKL), contributing to osteoclast differentiation and activation.^2^^,^^10^^,^^11^^,^^59^^,^^60^ (C) Chronic cytokine imbalance may, over time, contribute to effector T-cell differentiation and activation. Effector T-cell signaling and associated cytokine expression (eg, IL–17) may be responsible for the development of additional symptoms, such as skin involvement in SAPHO. Whether effector T-cells also contribute to bone inflammation/remodeling remains unknown but is likely because of pro-inflammatory cytokine release. Indeed, in animals, IL–17 signaling has been linked with increased osteoclastogenesis.^61^

As mentioned above, reduced IL–10 and IL–19 expression contributes to increased activation of the NLRP3 ((NOD)-like receptor family pyrin domain containing 3) inflammasome (Figure 4A).^46^^,^^47^^,^^54^ The NLRP3 inflammasome is a cytoplasmic multiprotein complex that plays a central role in innate immune responses against infectious pathogens and other “danger signals” (including ions, etc.).^55^ In response to its activation, NLRP3 joins with the apoptosis-associated speck-like adaptor molecule (ASC speck), facilitating conversion of inactive pro-caspase 1 to inflammatory caspase 1.^56^ As a result, NLRP3 inflammasome assembly results in the cleavage and activation of inactive pro-IL–1β and pro-IL–18 into their active forms IL–1β and IL–18.^47^ Notably, caspase 1 also mediates inflammatory cell death, so called pyroptosis, which contributes to the release of intracellular inflammatory components (including IL–1β, IL–18, assembled inflammasomes, etc.) that contribute to the spread of inflammation but also termination of ongoing pro-inflammatory cytokine release.^57^ Notably, in monocytes from CNO patients, reduced DNA methylation (another activating epigenetic mark) allows for increased RNA and, subsequently, protein expression of inflammasome components NLRP3 and ASC (encoded by the *PYCARD* gene), as well as IL–1β, which further promotes inflammatory cytokine activation and release.^47^^,^^52^ Whether this is a “primary” defect in CNO monocytes or the result of ongoing inflammation (as it is in the cryopyrin associated Muckle Wells syndrome^58^) currently remains unknown.

### P2X7, the “new kid on the block”

Recently, exome sequencing of a family with a history of CNO over 2 generations, we identified presumed gain-of-function variants in the *P2RX7* gene of affected individuals (mother and daughter).^50^ The P2X7 protein is an ATP-sensitive transmembrane receptor with ion channel function. Upon activation, P2X7 mediates sodium (Na^+^) and calcium (Ca2^+^) influx and potassium (K^+^) efflux (Figure 4A). Potassium efflux is a strong stimulus of NLRP3 inflammasome assembly and pyroptosis.^50^^,^^62^ Targeted sequencing of *P2RX7* in a national cohort of 191 CNO patients from Germany identified 70 SNPs; 5.8% of patients carried rare presumably damaging *P2RX7* variants (compared to 1.9% of 1874 healthy regional controls), and 32.4% of patients displayed rare lower impact P2RX7 variants (which compared to 4.4% of healthy controls). Notably, carriers of rare damaging *P2RX7* variants were more likely to require second-line treatments and displayed extra-skeletal manifestations, for example, abdominal symptoms and lymphadenopathy.^50^ Considering all rare variants in *P2RX7* (high- and low-impact), the *P2RX7* gene is >10 times more variable among CNO patients when compared to controls, underscoring its likely key role in its molecular pathophysiology.

Functional studies in primary human cells from CNO/CRMO patients and in genetically modified THP-1 monocytes carrying the index-case *P2RX7* variant c.349C > T/p.Arg117Trp/rs28360445 confirmed increased inflammasome assembly and associated pro-inflammatory cytokine release (namely IL–1β and especially IL–18) that was, however, accompanied by reduced pyroptosis. This is interesting and of potential key relevance as it may prolong cytokine release and allow cells to be restimulated. The CNO-associated rare *P2RX7* variant c.920G > A/p.Arg370Gln/rs28360457, based on in silico prediction, expectedly associated with loss-of-channel-function, altered inflammasome assembly and cytokine release, and prolonged cell survival. Notably, although *P2RX7* c.920G > A may associate with prolonged but low-level cytokine release, it occurred in combination with 2 more common predicted gain-of-function variants and may therefore act in concert with these.^50^ Supporting this hypothesis, rs28360457 has previously been linked with bone loss, osteoporosis, and increased risk of hepatocellular carcinoma, all conditions characterized by inflammation.^50^

### Potential mechanisms driving osteoclast activation

Until recently, it remained unclear why pathological inflammasome activation in monocytes from CNO patients results in bone pathology. Likely, a combination of mechanisms is responsible for the bone phenotype of CNO/CRMO. Greenhill and colleagues demonstrated in a murine-induced arthritis model that reduced IL–10 expression and subsequently increased inflammasome assembly and IL–1β release promote bone inflammation.^56^ Indeed, imbalanced pro- and anti-inflammatory cytokine expression contributes to increased engagement between RANK and its ligand (RANKL), thereby enhancing osteoclast differentiation and activation (Figure 4B).^2^^,^^10^^,^^11^^,^^59^^,^^60^ Another recently appreciated mechanism is linked to *P2RX7* gain-of-function variants. Osteoclast differentiation and activation have directly been linked with P2X7 activity, which further supports increased pathological bone remodeling in CNO.^50^ Lastly, a third mechanism through pathological adaptive immune activation appears possible.^18^^,^^63^ Ongoing pro-inflammatory cytokine release, likely in the context of tissue damage, may result in the differentiation and activation of IL–17 expressing effector T-cell populations (Figure 4C). To which extent effector T cells contribute to bone inflammation/remodeling remains unknown. However, IL–17 signaling has been linked with increased osteoclastogenesis and inflammatory bone loss.^61^ Furthermore, IL–17 signaling may not only promote osteo-inflammation but also result in the development of skin (namely psoriasis, palmoplantar pustulosis) and joint (arthritis) manifestations frequently associated with SAPHO.^18^^,^^63^ This hypothesis may explain why adults more frequently exhibit SAPHO (as compared to children who usually experience CNO without skin disease), due to ongoing (adaptive) immune activation.

### Serum protein signatures as future biomarkers

Although currently no widely accepted or prospectively tested biomarkers are available, preliminary studies suggest serum protein panels to discriminate between CNO and differential diagnoses.^64^^,^^65^ In the absence of diagnostic criteria, easily accessible diagnostic biomarkers are urgently needed to advance the clinical care of patients with CNO. A study using multiplex assay analysis of 25 inflammatory proteins from patients with CNO, “CNO-mimicker” conditions and healthy controls identified differences in monocyte-derived pro-inflammatory serum proteins across groups.^64^ Patients with CNO/CRMO exhibited higher serum levels of IL–6, IL–12, eotaxin, macrophage inflammatory protein-1β, regulated on activation, normal T-cell expressed and secreted protein), monocyte chemotactic protein 1, and the soluble IL–2 receptor (IL–2R) when compared to healthy controls. Distinguishing between patients with CNO and JIA/Crohn’s disease, however, was more challenging. Several serum proteins associated with treatment outcomes (defined by Paediatric CNO (PedCNO) scores^66^), including IL–6 and eotaxin.^64^ A subsequent study included preliminary biomarker candidates from above, the calcium-binding protein S100A8, and bone metabolism markers collagen Iα, osteopontin, osteoprotegerin, and osteonectin. Protein signatures were tested across CNO and additional “CNO-mimicker” conditions, including leukemia, lymphoma, osteoarticular infections, and reactive arthritis. Results largely confirmed previous reports with raised IL–6 in CNO patients when compared to all other groups, with MIP-1β, RANTES, IL–6, S100A8, sIL–2R, IL–1RA and collagen Iα raised in CNO sera as compared to healthy controls.^65^ ROC analysis suggested eotaxin, IL–6, RANTES, collagen Iα, and sIL–2Rα as potential diagnostic biomarkers, and eotaxin (>110 pg/mL) and IL–6 (>17 pg/mL) as a minimal panel (sensitivity 93%, specificity 97%).^65^ However, overlap existed between disease groups when considering proteins individually, and differences between CNO and mimicker conditions/healthy controls were small and yet to be validated in larger unrelated cohorts. Thus, their translation into clinical practice may be challenging. Experiments using unbiased mass spectrometry to identify disease-associated protein signatures in CNO are underway, and first results are awaited later in the year.

Taken together, inflammasome activation and cytokine dysregulation play pronounced roles in the molecular pathophysiology of CNO/CRMO. However, it remains unclear, which factors trigger disease expression and/or flares in (genetically) predisposed individuals. Factors defining disease phenotypes, such as patterns of skeletal lesions (mono vs multifocal, axial vs peripheral involvement) or additional organ involvement (skin disease, arthritis, gastrointestinal involvement, etc.), also remain to be determined.

### Currently available treatment

In the absence of a complete pathomechanistic understanding of CNO/CRMO, no available clinical trials, and/or regulatory approval, empiric immune modulating treatment is used to treat CNO/CRMO.^2^^,^^8^^,^^67^ Treatment and care are based on personal and institutional experience that are guided by case reports, retrospective case series,^4^^,^^5^^,^^14^^,^^17^^,^^68–70^ and (more recently) consensus treatment plans.^67^ Variable accessibility/funding across geographic regions significantly affect treatment choices (eg, TNFi are commonly used in continental Europe but are not widely accessible in the UK).^67^ Recently collated views from patients, clinicians, and academics confirm the urgent need for interprofessional collaboration to generate evidence and harmonize effective treatment (preferably through clinical trials) in CNO/CRMO and SAPHO.^8^^,^^71^^,^^72^ In line with this, the UK NIHR Clinical Studies Group for Paediatric Rheumatology published a research priority setting exercise that included clinical trials in CNO/CRMO among the top 9 disease-specific research priorities.^72^ Despite this, and independent of the constantly growing pathomechanistic understanding, to date, there has been only one randomized controlled open-label pilot study of pamidronate vs placebo in a small adult cohort of CNO/CRMO patients.^73^ Notably, none of the treatments discussed here are licensed for the treatment of CNO/CRMO.

Usually, ***nonsteroidal anti-inflammatory drugs (NSAIDs)*** are chosen as first-line treatment in patients with CNO in the absence of vertebral involvement. NSAIDs control prostaglandin E production through the inhibition of cyclooxygenases thereby affecting nociception.^10^ Because prostaglandin E is essential for osteoclast activity, NSAIDs have inhibitory effects on osteoclasts, thereby reducing pathological bone remodelling.^10^ The NSAIDs naproxen has been reported effective in >60% of CNO patients after a follow-up period of 12–18 mo in an open-label study.^66^ Although a retrospective case series confirms these observations, it also shows that >50% of patients will flare at a median of 29 mo, suggesting that most CNO patients will require more aggressive treatment and long-term follow-up.^4^

**Figure 5:**
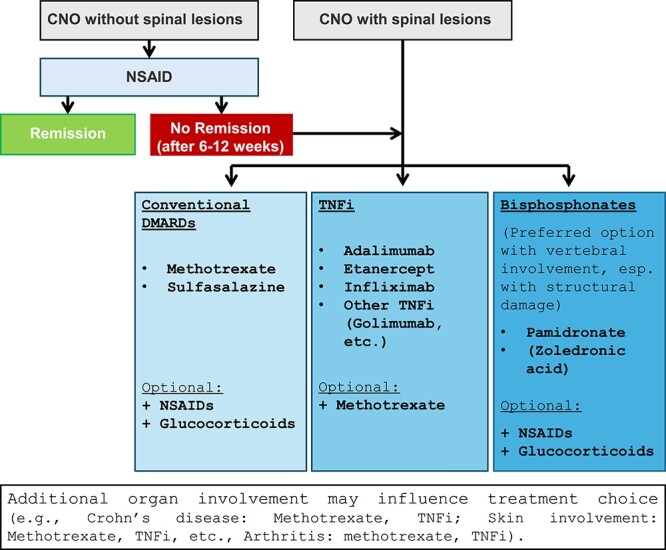
Currently used treatments in (pediatric) CNO. Most CNO patients will receive NSAIDs as first-line treatment (usually naproxen at 10–15 mg/kg and day in 2 doses). In patients who fail to respond sufficiently or in those with vertebral involvement, additional treatments should be considered. Notably, in patients with vertebral damage, especially in those with structural damage, bisphosphonates are the preferred choice. Experience with zoledronic acid is limited in pediatric CNO. Treatment choices should be reviewed to consider potentially necessary adjustment after 6-12 wk, in patients with vertebral involvement more closely.

In patients who fail to respond to NSAIDs or in those with vertebral involvement, additional treatment is considered necessary. The ***conventional disease-modifying-drugs (DMARDs)*** methotrexate and sulfasalazine affect cell proliferation, differentiation, and inflammatory cytokine release.^74^ Their use in CNO has been reported in few retrospective studies, suggesting limited effects on pain and bone inflammation^31^^,^^67^^,^^75^^,^^76^ (Figure 5).

***Bisphosphonates*** counteract osteoclast activity through inducing apoptosis, through their affinity for hydroxyapatite on bone surfaces.^77^ Although only one randomized trial exists in an adult CNO cohort,^73^ pamidronate is commonly used in the treatment of pediatric and adult CNO and has been shown to reduce proinflammatory cytokine expression^10^^,^^11^ (Figure 5). Pamidronate induces remission in a large proportion of patients with CNO/CRMO, including in vertebral disease.^10^^,^^44^^,^^78^^,^^79^ Two alternative treatment regimens have been reported for pamidronate that are commonly used in clinical practice: 1 ​mg/kg/dose (max. 60 mg/dose) every month (first dose max. 0.5 mg/dose) or 1 ​mg/kg/dose (max. 60 mg/dose) on 3 consecutive days every 3 mo for 9–12 mo.^30^

Recently, shortage of pamidronate across multiple countries has resulted in increased use of zoledronic acid to treat CNO.^80^ Zhao et al. first reported rapid response to treatment with zoledronic acid in combination with the TNFi infliximab in pediatric CNO.^81^ Several recent reports are available in the adult CNO cohort, suggesting beneficial effects on pain and local swelling (CNO^82^, SAPHO^33^^,^^83^). In CNO, zoledronic acid is usually used intravenously at 0.0125–0.025 mg/kg/dose (max. 4 mg) every 6–12 mo.^33^^,^^81–83^

The use of bisphosphonates has especially been considered beneficial in patients with vertebral involvement and structural damage.^10^^,^^30^ Recently, a retrospective multicenter study from Schnabel et al. demonstrated favorable effects of both bisphosphonate therapy and TNFi on bone inflammation (on MRI) and clinical symptoms, with bisphosphonates leading to quicker radiological resolution of lesions but TNFi therapy resulting in fewer disease flares.^84^ Although based on preliminary data from a retrospective multicenter cohort, this may argue for first-line treatment with pamidronate in CNO patients with vertebral involvement, followed by TNFi maintenance therapy (see below).

Because of concerns around side-effects and the long biological half-life of bisphosphonates, they are usually considered for patients refractory to other treatments or with primary vertebral involvement (especially in the presence of structural damage).^10^ Main concerns are acute phase reaction with influenza-like symptoms (that can be masked with, eg, paracetamol),^85^ growth retardation (notably, based on data from rat models),^86^ and osteonecrosis (of the jaw),^10^ which has been reported in adults receiving bisphosphonates in the context of cancer treatment and subsequent dental procedures.^87^ Reports of pamidronate-associated osteonecrosis in children are rare, and no case of bisphosphonate-associated osteonecrosis has been reported in CNO.^88^ Because zoledronate is significantly (approximately 100-fold) more potent when compared to pamidronate,^89^ side effects may be more common and more pronounced. Common influenza-like symptoms but also rare complications (such as seizures^90^) have to be considered and monitored. However, a recent case series and literature review comparing safety and efficacy of pamidronate and zoledronate in pediatric CNO did not report differences between treatments.^91^

In addition to bisphosphonates, osteoclast activity can be inhibited by blockade of RANKL with the recombinant RANKL inhibitor denosumab, which may promise future potential in the treatment of CNO. However, at this point, there are no published reports on successful application of these treatment options in pediatric or adult CNO.^10^

**Figure 6:**
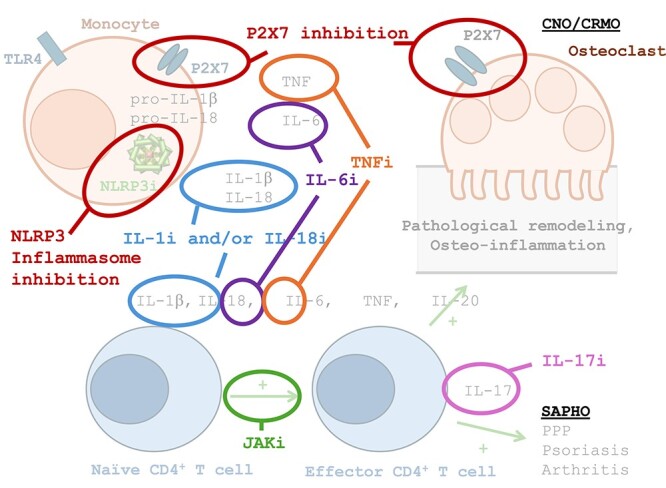
Available and future targeted treatments in CNO. Based on the growing pathomechanistic understanding of CNO discussed in this article, target-directed interventions may be possible to treat patients. A pronounced dysregulation of pro- and anti-inflammatory cytokines has been observed in monocytes from CNO patients. Thus, cytokine blocking strategies appear particularly promising.^71^ TNF inhibitors (including adalimumab, infliximab, etanercept) are already used for several inflammatory conditions associated with CNO/CRMO including inflammatory bowel disease, inflammatory arthritis, and psoriasis and were reported beneficial in most CNO/CRMO patients.^4^^,^^84^ The pro-inflammatory cytokine IL–18 belongs to the IL–1 cytokine family. Uncontrolled IL–1β and IL–18 release are key features of CNO. As mentioned above, CNO-associated variants in *P2XR7* promote IL–1β and IL–18 activation and release from monocytic cells.^50^ Selective IL–1β and IL–18 inhibitors have been developed but have not found their way into routine treatment of CNO.^95^^,^^96^ Case series, however, promise potential for IL–1 blockade in CNO.^97^ Interleukin-6 levels are elevated in the serum/plasma of adult and pediatric patients with CNO/CRMO and SAPHO.^49^^,^^64^^,^^92^^,^^98^^,^^99^ The blocking IL–6 receptor antibody tocilizumab is a therapeutic option licensed for polyarticular JIA, RA, and other diseases.^100^ However, data on the use in CNO are limited and not conclusive.^101^^,^^102^ Recent studies highlight P2X7 as a potentially interesting target in CNO.^50^ Thus, P2X7 blockade may be a therapeutic option in inflammatory disease, including CNO. The NLRP3 inflammasome is activated by P2X7 and plays a key role in a wide variety of diseases including CNO.^103^ Although (currently) not for use in humans, the small molecule MCC950 inhibits NLRP3 inflammasome assembly and subsequent IL–1β release in whole blood from humans.^104–106^ The apoptosis-associated speck-like protein (ASC) associates with NLRP3 and pro-caspase-1 to form the NLRP3 inflammasome.^11^ Recent work showed increased ASC speck formation in cells with WT cells P2RX7 expression vs controls.^50^ A small molecule inhibitor of ASC, MM01 has been studied in vitro, showing inhibition of ASC-dependent inflammasomes.^107^ Based on these observations, small molecule inhibitors targeting the NLRP3 inflammasome may be promising future therapeutic considerations.^108^^,^^109^ The JAK pathway is central for the regulation of innate cytokine expression.^110^ Therapeutic blockade of the JAKs may control the expression of inflammatory interleukins such as IL–6, IL–17, or IL–23.^111^ Indeed, tofacitinib has shown promising results in SAPHO, including one open-label prospective observational study.^112^ Notably, none of the here discussed treatments are licensed for the use in CNO (PPP: Palmoplantar pustulosis).

As mentioned above, dysregulation of pro- and anti-inflammatory cytokines has been observed in monocytes from CNO patients.^46^ Preclinical research identified several possible targets for treatment, with pro-inflammatory cytokines IL–1 and TNF as promising candidates. Thus, ***cytokine blocking strategies*** are of particular interest to clinicians in the field^71^ (Figures 5 and 6). Pro-inflammatory IL–1β regulates the expression of TNF that has been found to be elevated in the plasma of CNO patients when compared to healthy controls.^92^ TNF inhibitors (including adalimumab, infliximab, etanercept) are used for several inflammatory conditions associated with CNO/CRMO including IBD, inflammatory arthritis, and psoriasis. However, TNFi is currently not licensed for the treatment of CNO/CRMO. Efficacy of TNFi in CNO/CRMO has been suggested by several groups. Schnabel and colleagues reported beneficial and long-lasting effects of TNFi in most CNO/CRMO patients.^4^^,^^84^ Kostik et al. reported improvement of vertebral CNO lesions after pamidronate therapy was unsuccessful, inducing remission in 38/53 (73%) children.^93^ Similar results were seen in a small retrospective study, with 5/11 (46%) CNO patients treated with TNFi reaching clinical remission, and 10/11 (91%) showing “some improvement.”^94^ The European “Eurofever” registry collected data from 496 pediatric and adult CNO/CRMO patients, 34 of whom received TNFi treatment (remission rates 40%–50%).^69^

**Table 2:** Expert consensus for a proposed clinical trial in CNO.^71^

**Population**	
	Inclusion: CNO patients diagnosed by an expert pediatric rheumatologist, fulfilling (awaited) ACR/EULAR classification criteria Age: 2-18 yr Unifocal or multifocal disease Failure to respond to NSAIDs in the absence of vertebral involvement Conventional/biological DMARD and pamidronate naïve Exclusion criteria: Atypical/complicated CNO Skin involvement requiring systemic treatment
**Intervention/comparator**	
	Biologic DMARDs are the group of medications that promise efficacy and safety in the treatment of CNO: IL–1 blocking strategies Interleukin-17 blocking strategies As placebo use appears unethical in the presence of good cohort data suggesting efficacy and safety of empiric treatment, the bisphosphonate pamidronate should be used as comparator.^3^^,^^82^
**Primary outcomes**	
	Patient pain visual analogue scale (VAS) Physician global (VAS) Patient global (VAS)
**Secondary outcomes**	
	PedCNO score (includes: ESR, number of radiological lesions, physician global, patient/parent global, CHAQ)^74^ Improvement on MRI (number and/or signal intensity/size of lesions)

Abbreviations: ACR/EULAR, American College of Rheumatology/European Alliance of Associations for Rheumatology; CHAQ, Childhood Health Assessment Questionnaire; CNO, chronic nonbacterial osteomyelitis; DMARDS, Conventional disease-modifying antirheumatic drugs; ESR, erythrocyte sedimentation rate; MRI, magnetic resonance imaging; NSAIDs, nonsteroidal anti-inflammatory drugs; VAS, visual analogue scale

Paradoxical psoriasis is a known side effect of TNFi.^23^ In CNO/CRMO, preliminary results suggest that paradoxical psoriasis is more common in CNO patients receiving TNFi when compared to “other” CNO patients.^113^ The fact that CNO/CRMO is per se associated with psoriasis, however, complicates data interpretation. Although also patients treated with the bisphosphonate pamidronate can develop psoriasis, one study suggested that this may be slightly more common among patients treated with TNFi.^113^ Recently, Yang et al. conducted a retrospective analysis of 18 patients with CNO treated with golimumab after failing treatment with other TNFi. CNO patients treated with golimumab showed improvement in ESR, MRI findings, and Physician Global Assessment score. Paradoxical psoriasis from previous TNFi use also improved in 9/18 (50%) patients.^114^ Notably, switching treatment to an alternative TNFi has previously been suggested as an approach to paradoxical psoriasis.^115^

### Future directions

Based on recent pathomechanistic studies, ***additional cytokine blockers*** promise potential for the use in CNO/CRMO. Increased IL–1β expression and release have been observed in human CNO/CRMO patients and murine disease models^116–118^ (Figure 6).

***Therapeutics to target IL–1β*** include anakinra (recombinant IL–1 receptor antagonist),^119^ canakinumab (IL–1β specific blocking antibody),^120^ and rilonacept (a fusion protein consisting of the ligand-binding domains of the human IL–1 receptor linked the Fc region of human IgG1).^121^

A case series from Italy reported beneficial effects of anakinra in a complex CNO cohort of patients who previously failed NSAIDS, bisphosphonates, and/or glucocorticoids.^97^ Although only a proportion of patients responded to anakinra, a comparable low dose of anakinra (1-2 mg/kg) was used in patients failing other treatment options. Thus, anakinra or other IL–1 blockers may be more efficient when used earlier in the disease course and at a high enough dose.^97^

A case report of a 13-yr-old girl reported clinical, biochemical, and radiological resolution of CNO associated with pyoderma gangrenosum in response to canakinumab.^122^ Furthermore, 2 brothers with Majeed syndrome were successfully treated with canakinumab, following unsuccessful treatment attempts with the TNFi etanercept.^123^

Rilonacept has not been trailed in patients with “classical” sporadic CNO but has been successfully used in patients with DIRA.^124^

The pro-inflammatory cytokine IL–18 belongs to the IL–1 cytokine family. It is implicated in IBD and rheumatoid arthritis.^125^^,^^126^ Uncontrolled IL–18 release is the key feature of autoinflammatory systemic JIA,^127^^,^^128^ macrophage activation syndrome,^128^^,^^129^ and NLRC4-associated autoinflammatory disease.^130^^,^^131^ As mentioned above, CNO-associated variants in *P2XR7* promote IL–18 activation and release from monocytic cells.^50^ Selective IL–18 inhibitors have been developed but have not found their way into routine treatment yet.^95^^,^^96^

***IL–6*** is a cytokine with a broad spectrum of biological functions in inflammation, immunity, hematopoiesis, and disease.^132^^,^^133^ It induces the synthesis of acute phase proteins and antibodies.^132^ Because inflammasome activation and subsequent IL–1 signaling promote IL–6 expression, IL–6 may be a promising treatment target in CNO/CRMO. Indeed, IL–6 levels are elevated in the serum/plasma of adult and pediatric patients with CNO/CRMO and SAPHO.^49^^,^^64^^,^^92^^,^^98^^,^^99^ The blocking IL–6 receptor antibody tocilizumab is a therapeutic option licensed for polyarticular JIA, RA, and other diseases.^100^ Three published reports are available, suggesting beneficial effects in CNO/CRMO: 2 patients with adult-onset CNO with tocilizumab, and in combination with methotrexate in one child; however, reported use in 2 SAPHO patients did not corroborate these findings.^101^^,^^102^ However, overreporting of successful use may play a role, and it remains unclear what proportion of CNO/CRMO patients may benefit from IL–6 blockade.

The ***JAK pathway*** is central for the regulation of innate cytokine expression, directing subsequent adaptive immune response and confining inflammatory responses.^110^ Therapeutic blockade of the JAKs may control the expression of inflammatory ILs such as IL–6, IL–17, or IL–23.^111^ Indeed, tofacitinib has shown promising results in SAPHO, including one open-label prospective observational study.^112^ Only one small case series of 4 patients is available reporting mixed responses in pediatric CNO/CRMO. Two patients (50%) showed radiological and clinical improvement, while one patient could not tolerate treatment because of gastrointestinal side-effects despite improvements in CNO symptoms, and one experienced worsening CNO/CRMO.^70^

Recent studies highlight the ***P2X7 receptor*** as a potentially interesting target in CNO/CRMO.^50^ P2X7 inhibition had previously been discussed for the treatment of osteoarthritis,^134^ and McInnes et al. demonstrated that P2X7 blockade reduces joint inflammation in rats.^135^ Positive results of P2X7 blockade were also seen in murine models of lupus nephritis, diabetic retinopathy, and tuberculosis.^136^^,^^137^ Recently, P2X7 inhibition delivered promising results in the treatment of depression in humans.^138^ Taken together, P2X7 blockade may be a therapeutic option in inflammatory disease. However, data are very limited, and clinical trials are urgently needed.

The ***NLRP3 inflammasome*** plays a key role in a wide variety of diseases including in cancer, metabolic disease, infection, aging, and autoinflammatory disease including CNO/CRMO^103^ (Figure 6). In a substantial proportion of CNO/CRMO patients (approximately 40%), P2X7 dysregulation contributes to increased inflammasome activation and may be targeted therapeutically.^50^ However, additional mechanisms may be involved and remain only partially understood.^46^^,^^47^ Although (currently) not available for use in humans, the small molecule MCC950 inhibits NLRP3 inflammasome assembly and subsequent IL–1β release in whole blood from humans,^104–106^ which promises potential for future therapeutic considerations. The apoptosis-associated speck-like protein (ASC) associates with NLRP3 and pro-caspase-1 to form the NLRP3 inflammasome.^11^ Recent work showed increased ASC speck formation in human monocyte cell lines (THP-1) expressing CNO-associated P2X7 variants when compared to wild-type controls.^50^ A small molecule inhibitor of ASC, MM01 has been studied in vitro, showing inhibition of ASC-dependent inflammasomes, and improved clinical outcomes in murine models of inflammasome-induced peritonitis.^107^ Based on these observations, small molecule inhibitors targeting the NLRP3 inflammasome assembly (eg, through blocking NLRP3, ASC) may be promising future therapeutic considerations. Indeed, several companies are currently investing in this area.^108^^,^^109^

Regardless of many attempts through industry contacts or academic funding, clinical trials are currently not available for CNO/CRMO. Concerns of potential funders are focused on the rarity of the condition and limited financial gain for pharmaceutical industry considering expiring or expired patents of some treatment candidates. However, clinical trials are a shared priority for patients, families, and clinicians caring for CNO/CRMO patients as they would allow for treatment licensing and its access to wide patient groups.^8^^,^^72^ During an international meeting on CNO/CRMO and autoinflammatory bone disorders in Liverpool (2022), expert consensus (involving leading clinicians, academics, and patient representatives) has been achieved suggesting inclusion and exclusion criteria, as well as preliminary outcomes for a clinical trial^71^ (Table 2). Furthermore, ACR/EULAR classification criteria and outcome measures generated by the OMERACT group are awaited.^139^

## Conclusions

In the absence of diagnostic criteria and/or disease biomarkers, CNO/CRMO remains a diagnostic challenge. Treatment is empiric and targets inflammation and bone remodeling. Recent progress in understanding the involvement of pathological inflammasome activation and associated cytokine release promises exploitation of knowledge to allow for clinical trials of targeted treatments (cytokine blockade, P2X7 inhibition, etc.) and the development of biomarkers for the diagnosis and treatment monitoring. Clinical trial design will be informed by imminently awaited classification criteria and outcome measures.

## Data Availability

This review does not include primary data.
